# Case Report: Rare case of primary rectal adenocarcinoma presenting as a smooth submucosal elevation: endoscopic submucosal excavation and 4 years and 11 months follow-up

**DOI:** 10.3389/fmed.2026.1777158

**Published:** 2026-03-02

**Authors:** Qing-li Kong, Yao-wen Zhang, Huan-qing Fan

**Affiliations:** 1 Phase I Clinical Trial Laboratory, Affiliated Hospital of Jining Medical University, Jining, China; 2 Department of Endoscopy, Affiliated Hospital of Jining Medical University, Jining, China; 3 Department of Rehabilitation Medicine, Affiliated Hospital of Jining Medical University, Jining, China

**Keywords:** clinical cure, endoscopic submucosal excavation (ESE), primary adenocarcinoma, smooth surface, submucosal elevation

## Abstract

Primary adenocarcinoma of the rectum presenting as a smooth submucosal elevation is clinically rare. Due to its insidious symptoms and diagnostic challenges in imaging differentiation, the malignant risk is easily overlooked. We report a 64-year-old female patient who was incidentally found to have a 0.9 cm diameter submucosal elevation in the rectum (15 cm from the anus) during physical examination. Endoscopic ultrasonography (EUS) revealed a hypoechoic mass originating from the muscularis mucosae. Based on intraoperative ultrasound examination, a preliminary diagnosis of rectal neuroendocrine tumor (NET) is made. The lesion was completely resected via endoscopic submucosal excavation (ESE), and postoperative pathology confirmed adenocarcinoma with negative resection margins. The patient had an uneventful postoperative recovery and was followed up for 4 years and 11 months, nearly 5 years without tumor recurrence, metastasis, or impairment of intestinal function, maintaining a good quality of life. This case highlights the necessity of vigilant evaluation for smooth submucosal rectal lesions to avoid missed diagnosis of malignancy. As an advanced minimally invasive endoscopic technique, ESE enables complete resection of submucosal lesions with the advantages of minimal trauma, rapid recovery, preserved intestinal function, and favorable long-term safety, providing valuable clinical experience for the management of similar rare cases.

## 1 Introduction

Most rectal neuroendocrine tumors are early-stage, small-sized, and superficial lesions. Primary rectal adenocarcinoma typically presents as mucosal roughness, erosion, ulceration, or cauliflower-like protrusion, whereas adenocarcinoma manifesting as a smooth submucosal elevation is extremely uncommon (1). Owing to its submucosal location and intact overlying mucosa, this subtype typically lacks obvious clinical symptoms in the early stage and is often detected incidentally during routine physical examinations or workups for other diseases (2). The patient in this case was asymptomatic, and the lesion was identified solely through a routine check-up. In clinical practice, most patients with similar lesions are managed with observational follow-up (3). However, based on our clinical experience, further pathological examination is imperative to confirm the diagnosis and avoid overlooking malignant risks simply due to the “smooth surface” of the lesion. For such patients, endoscopic submucosal excavation (ESE) is recommended, as this procedure not only enables definitive diagnosis but also achieves endoscopic treatment. Notably, through long-term follow-up, we report for the first time that this patient has achieved clinical cure, highlighting the potential of ESE as an effective therapeutic strategy for this specific subtype of rectal adenocarcinoma.

## 2 Case description

**Figure 1:**
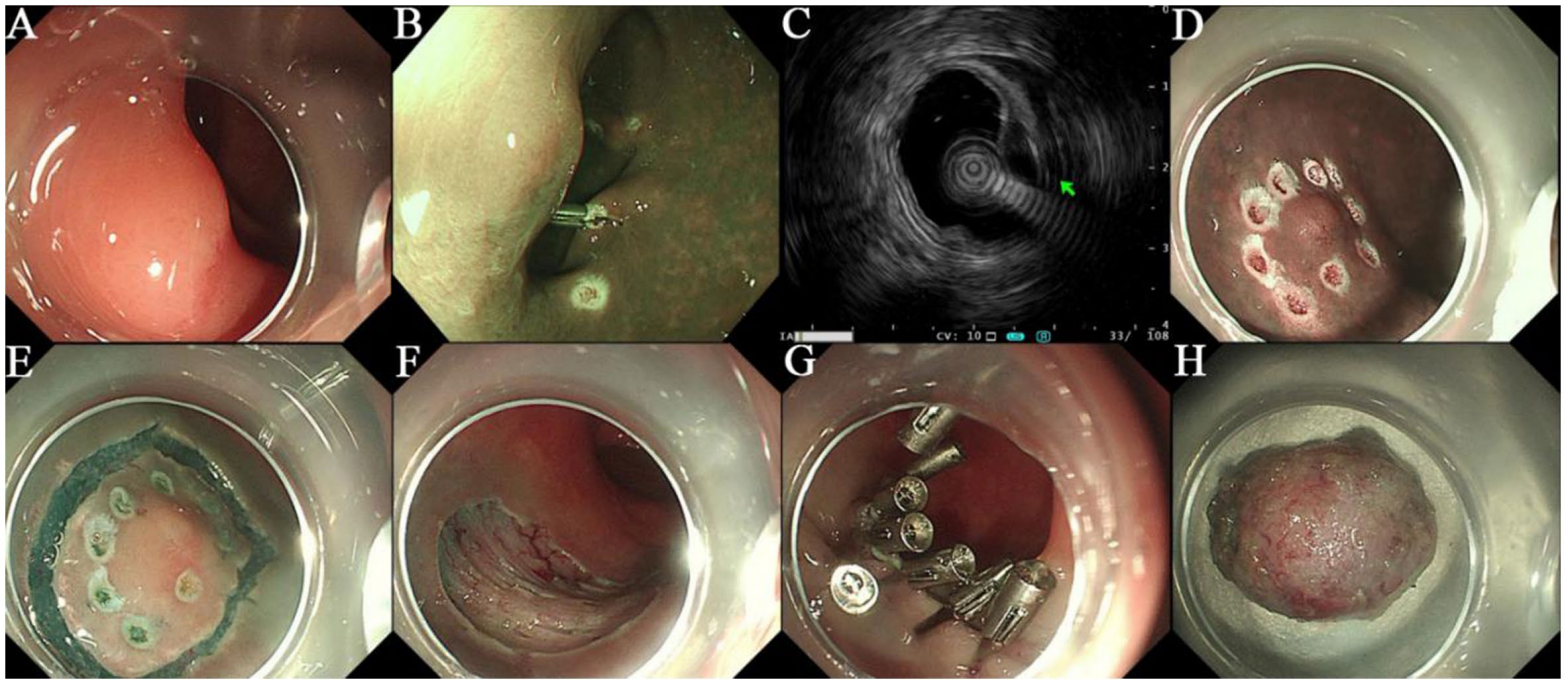
**(A)** The rectal protruding lesion showed a smooth surface under white light endoscopy. **(B)** No obvious large abnormal blood vessels were observed under narrow-band imaging (NBI). **(C)** Mini-probe endoscopic ultrasound (EUS) revealed a hypoechoic mass located in the muscularis mucosae, with clear borders, uniform internal echo, and a size of approximately 9.5 mm × 3.4 mm. **(D)** The area around the protruding lesion was marked. **(E)** The mucosal layer was circumferentially incised at a distance of about 5 mm from the lesion. **(F)** After complete resection of the lesion, the muscularis propria was fully exposed at the wound site. **(G)** The wound was closed using multiple metal clips (Micro-Tech, ROCC-D-26-195). **(H)** The completely resected mass was obtained from the submucosal plane.

**Figure 2:**
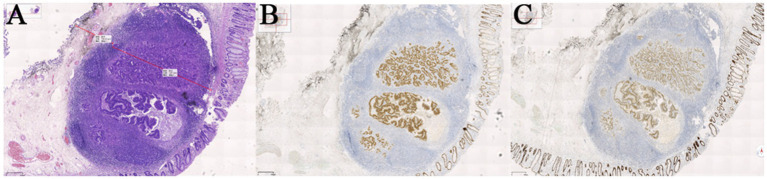
**(A)** HE staining, 40×. The long line indicates that the cancerous tissue is located 1.97 mm in the submucosa. The short line indicates that the cancerous tissue is 0.75 mm away from the basal resection margin. **(B)** IHC (InVision two-step method), CDX-2(+), 40×. **(C)** IHC (InVision two-step method), SATB-2(+), 40×. Pathological diagnosis: Adenocarcinoma (moderately to poorly differentiated) is identified in the submucosal hyperplastic lymphoid tissue of the rectum. Immunohistochemically, the tumor cells show diffuse strong positivity for CDX-2, CK20, and SATB-2, suggesting an intestinal origin. Tumor budding is graded as G1. No definite lymphovascular tumor emboli or perineural invasion is identified. No carcinoma is found at the lateral and basal resection margins. Immunohistochemistry: Tumor cells: CK(+), CK20(+), CDX-2(+), SATB2(+), CD56(−), CgA(−), Syn(−), P53 (wild-type), Ki-67 (+, >75%).

**Figure 3:**
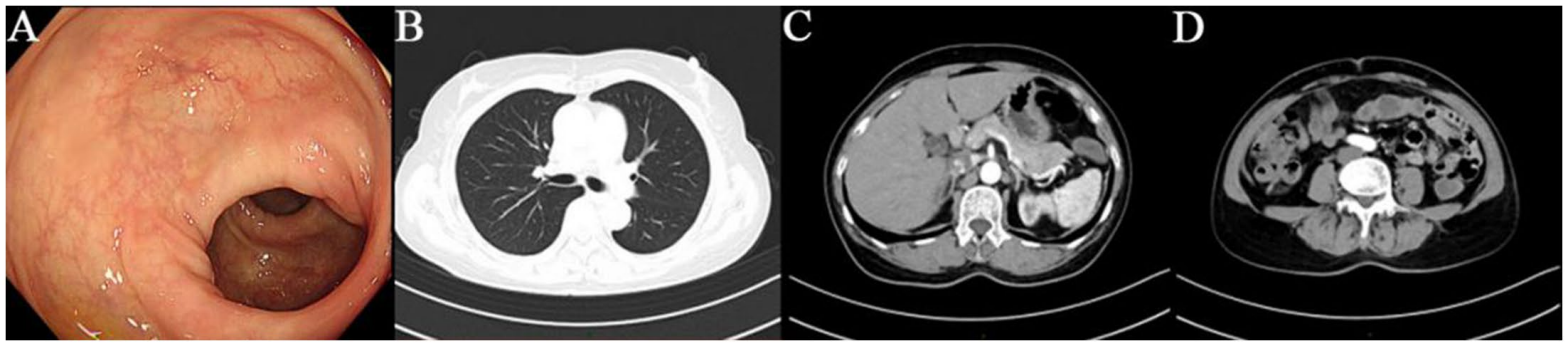
**(A)** Follow-up colonoscopy showed smooth and unremarkable mucosa. **(B–D)** Follow-up chest computed tomography (CT) and abdominal CT revealed no signs of recurrence or metastasis.

**Figure 4:**
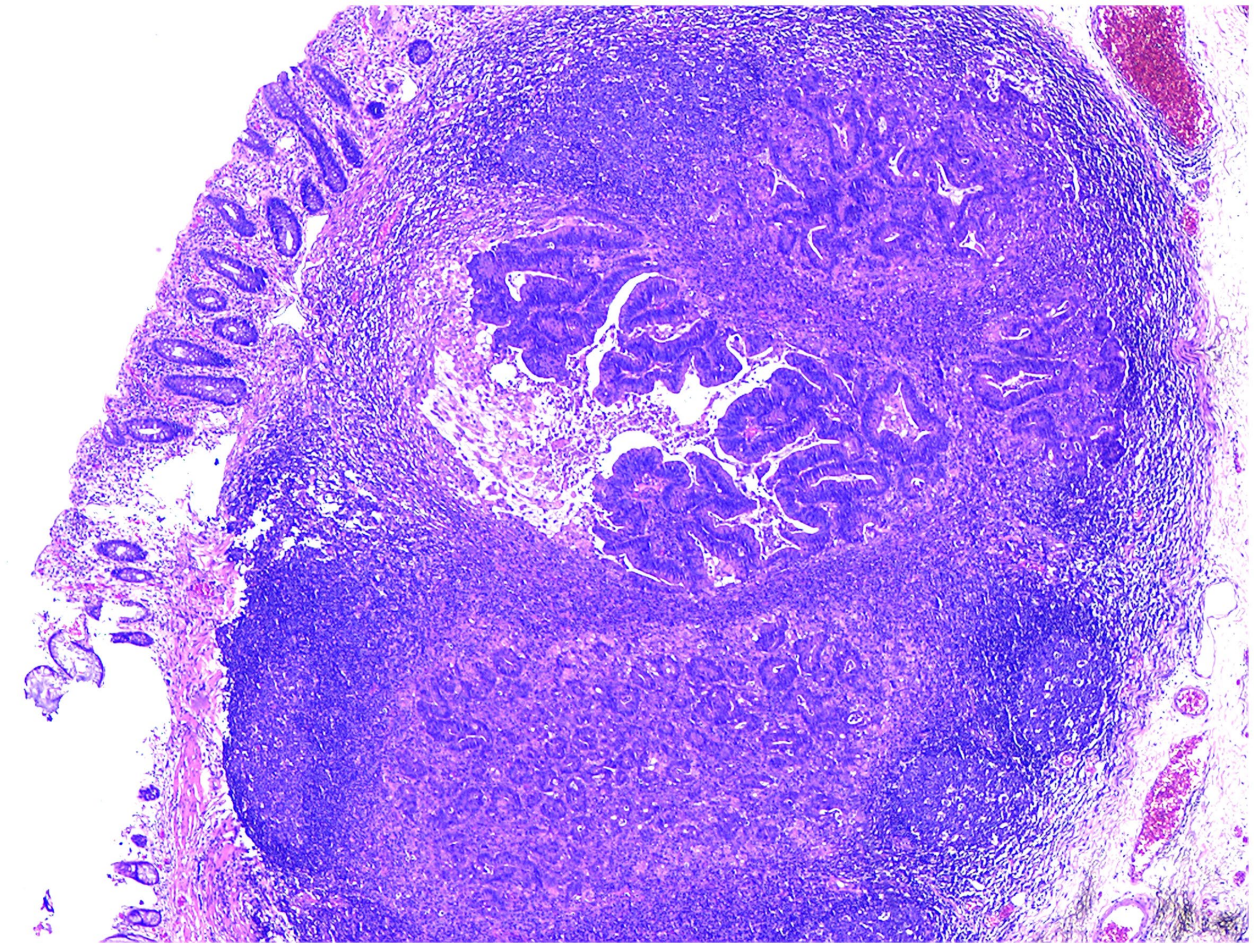
Adenocarcinoma was identified in the hyperplastic submucosal lymphoid tissue of the resected rectal specimen.

A 64-year-old female was admitted to our hospital with a 10-day history of “discovered rectal submucosal elevation.” Routine laboratory tests upon admission showed no abnormalities. On March 11, 2021, she underwent ESE for the rectal elevation and polypectomy under anesthesia. Colonoscopy revealed the endoscope reached 5 cm from the terminal ileum, with normal ileal mucosa, ileocecal valve, and appendix orifice. During withdrawal, a 0.4 cm hyperemic mucosal elevation in the ascending colon was ablated by argon plasma coagulation (APC). Six scattered mucosal elevations (0.2–0.5 cm in diameter), some with hyperemia, were detected in the sigmoid colon and rectum, all treated with APC; one local wound was closed with a metal clip. A 0.9 cm smooth submucosal elevation was found in the rectum (15 cm from the anus). EUS showed a 9.5 mm × 3.4 mm hypoechoic mass in the muscularis mucosae with clear borders and homogeneous internal echoes. Intraoperative diagnosis was rectal NET and multiple colorectal polyps. Treatment procedure: Submucosal injection was performed around the rectal lesion, followed by marginal pre-incision with a dual-knife. The lesion was completely excised by repeated submucosal injection and stepwise dissection, and the specimen was sent for pathological examination. Hemostasis was achieved at the wound, which was closed with 7 metal clips (Nanjing MicroPort Science & Technology Co., Ltd., soft tissue clip, model ROCC-D-26-195, batch number 200119238) (Figure 1). The patient recovered uneventfully. The postoperative pathology report was shown in Figure 2. During 4 years and 11 months, nearly 5 years follow-up, the patient had no tumor recurrence or metastasis (Figure 3), with normal defecation and good quality of life (see Figure 4).

## 3 Discussion

Traditionally, surgical resection was the mainstay of treatment for rectal submucosal lesions, but it is associated with large trauma, slow recovery, and potential impairment of intestinal function (4, 5). With the advancement of endoscopic technology, ESE has become an important therapeutic option for submucosal lesions originating from the muscularis propria. Its feasibility and advantages in this case are demonstrated as follows: ① Complete lesion resection: Through submucosal injection, incision, and dissection, ESE allows for en bloc resection of lesions arising from the muscularis propria under direct vision. Postoperative pathology confirmed negative resection margins with no residual tumor, achieving curative resection. ② Minimally invasive with rapid recovery: ESE is performed via natural orifices without laparotomy or laparoscopy, resulting in minimal intraoperative bleeding. The patient was discharged 3 days postoperatively, significantly shortening hospital stay and reducing medical costs. ③ Preservation of intestinal function: ESE only removes the lesion and a small amount of surrounding mucosa and submucosa without damaging the normal intestinal wall structure (6–10). Postoperatively, the patient had normal defecation without intestinal dysfunction such as abdominal pain or bloating, maintaining a good quality of life. The 4 years and 11 months, nearly 5 years follow-up showed no tumor recurrence or long-term complications such as intestinal perforation or stricture, confirming the long-term safety and efficacy of ESE in treating such lesions. Early colorectal cancer generally refers to histologically confirmed tumors with a maximum invasion depth not exceeding the superficial submucosa (1/3, sm1), and without other high-risk histological features, including poor differentiation, lymphatic or vascular invasion, and high-grade tumor budding (11). Precise and standardized histological risk assessment is required before and after endoscopic treatment to identify the presence of these high-risk features. In patients with two or more high-risk factors, the risk of lymph node metastasis exceeds 10%, and the curative effect of endoscopic resection is insufficient; therefore, additional surgery is recommended. For patients with a single controllable high-risk factor—such as isolated deep submucosal invasion (sm2 stage) without other high-risk features [well-differentiated, no lymphovascular invasion (LVI), G1 tumor budding], combined with a lesion diameter ≤20 mm and a superficial location (e.g., mid-to-lower rectum)—endoscopic treatment can be the first choice, accompanied by rigorous follow-up (12). Chen et al. (13) reported that poor histological grade and LVI are independent risk factors for lymph node metastasis. Their study indicated that the coexistence of poor histological grade and LVI may exert a synergistic effect, as patients with both high-risk factors exhibited significantly reduced survival rates. In contrast, a cohort study by Gijsbers et al. (14) identified tumor budding as a strong risk factor for lymph node metastasis. The author also emphasized that clinical treatment strategies should balance the risk of tumor recurrence against the risk of treatment-related complications. In addition, patients undergoing endoscopic treatment require comprehensive oncological follow-up to enable early detection and intervention of tumor recurrence. The goals of endoscopic treatment include complete resection confirmed by endoscopic evaluation, R0 resection with negative lateral and vertical margins, and curative resection without the aforementioned high-risk features (poor differentiation, lymphatic or vascular invasion, high-grade tumor budding) (15). Consistent with previous reports, our case exhibited G1 tumor budding, with no perineural or vascular invasion, and was negative for the above high-risk factors. Notably, the tumor in this case originated in the submucosa, which is distinct from tumors invading into the submucosa. After multidisciplinary team discussion and in accordance with the patient’s preference, a supplementary PET-CT scan performed within 3 months postoperatively showed no abnormal findings. Subsequent repeated PET-CT scans revealed no evidence of recurrence or metastasis, and regular follow-up contrast-enhanced chest and abdominal CT scans also showed no signs of recurrence or metastasis. Furthermore, telephone follow-up regarding the patient’s dietary and bowel habits indicated no discomfort. Although no similar cases have been reported in the literature, we adopted the management strategy for early invasive colorectal cancer, achieving complete resection, negative endoscopic margins, precise histological risk assessment, and close follow-up, which ensured the patient’s high-quality survival. In this case of smooth submucosal elevation-type primary rectal adenocarcinoma, EUS clarified the lesion origin and nature, and ESE achieved complete resection with negative margins. The patient had an uneventful recovery without severe complications, no tumor recurrence during 4 years and 11 months, nearly 5 years follow-up, and maintained a good quality of life. This case confirms that for early-stage, non-metastatic smooth submucosal rectal adenocarcinoma, ESE is a safe and effective minimally invasive treatment option, providing important clinical evidence for the management of similar rare lesions.

### 3.1 Patient perspective

The patient was discharged on the 3rd postoperative day without experiencing any adverse symptoms such as bleeding. She expressed sincere gratitude to the medical team, commenting that she was fortunate to have followed the physicians’ recommendation to undergo endoscopic submucosal excavation (ESE). Up to the present follow-up, no abnormalities have been identified, and her bowel habits as well as daily routines have remained consistent with the preoperative state, free of any discomfort. She also conveyed a reminder to patients with analogous conditions: early detection, timely diagnosis, and prompt treatment are the cornerstones of achieving an optimal prognosis.

## Data Availability

The original contributions presented in the study are included in the article/supplementary material, further inquiries can be directed to the corresponding authors.

## References

[1] Panzuto F O’Toole D Klöppel G Knigge UP Krejs GJ Tsoli M . Controversies in NEN: an ENETS position statement on the endoscopic management of localised gastric, duodenal and rectal neuroendocrine neoplasms. J Neuroendocrinol. (2025) 37:e70060. doi: 10.1111/jne.70060, 40524475 PMC12678012

[2] Kimura CMS Kawaguti FS Nahas CSR Marques CFS Segatelli V Martins BC . Long-term outcomes of endoscopic submucosal dissection and transanal endoscopic microsurgery for the treatment of rectal tumors. J Gastroenterol Hepatol. (2020) 36:1634–41. doi: 10.1111/jgh.15309, 33091219

[3] Kashida H Kudo SE. Early colorectal cancer: concept, diagnosis, and management. Int J Clin Oncol. (2006) 11:1–8. doi: 10.1007/s10147-005-0550-5 16508722

[4] Knol J Keller DS. Total mesorectal excision technique-past, present, and future. Clin Colon Rectal Surg. (2020) 33:134–43. doi: 10.1055/s-0039-3402776, 32351336 PMC7188504

[5] van der Heijden JAG Koëter T LJH S Sietses C Tuynman JB Maaskant-Braat AJG . Functional complaints and quality of life after transanal total mesorectal excision: a meta-analysis. Br J Surg. (2020) 107:489–98. doi: 10.1002/bjs.11566, 32154594 PMC7155085

[6] Santos JO Miyajima N Carvalho R Leal RF de Lourdes Setsuko Ayrizomo M Coy CSR. Feasibility of endoscopic submucosal dissection for gastric and colorectal lesions: initial experience from the Gastrocentro—UNICAMP. Clinics. (2013) 68:141–6. doi: 10.6061/clinics/2013(02)oa04, 23525307 PMC3584284

[7] Probst A Golger D Anthuber M Märkl B Messmann H. Endoscopic submucosal dissection in large sessile lesions of the rectosigmoid: learning curve in a European center. Endoscopy. (2012) 44:660–7. doi: 10.1055/s-0032-1309403, 22528673

[8] Spychalski M Dziki A. Safe and efficient colorectal endoscopic submucosal dissection in European settings: is successful implementation of the procedure possible? Dig Endosc. (2014) 27:368–73. doi: 10.1111/den.12353, 25181427

[9] Barret M Lepilliez V Coumaros D Chaussade S Leblanc S Ponchon T . The expansion of endoscopic submucosal dissection in France: a prospective nationwide survey. United European Gastroenterol J. (2017) 5:45–53. doi: 10.1177/2050640616644392, 28405321 PMC5384549

[10] Kawaguti FS Nahas CS Marques CF Martins BC Retes FA Medeiros RS . Endoscopic submucosal dissection versus transanal endoscopic microsurgery for the treatment of early rectal cancer. Surg Endosc. (2013) 28:1173–9. doi: 10.1007/s00464-013-3302-z, 24232053

[11] Přemysl F. Endoscopic treatment of early colorectal cancer. Vnitr Lek. (2022) 68:355–62. doi: 10.36290/vnl.2022.075, 36316196

[12] Přemysl F Jana Z Ondřej U. Endoscopic full-thickness resection versus endoscopic submucosal dissection in the treatment of colonic neoplastic lesions ≤30 mm-a single-center experience. Surg Endosc. (2021) 36:2062–9. doi: 10.1007/s00464-021-08492-0, 33860350 PMC8847190

[13] Chen PC Kao YK Yang PW Chen CH Chen CI. Long-term outcomes and lymph node metastasis following endoscopic resection with additional surgery or primary surgery for T1 colorectal cancer. Sci Rep. (2025) 15:2573. doi: 10.1038/s41598-024-84915-x, 39833323 PMC11747555

[14] Gijsbers KM Laclé MM Elias SG Backes Y Bosman JH van Berkel AM . Full-thickness scar resection after R1/Rx excised T1 colorectal cancers as an alternative to completion surgery. Am J Gastroenterol. (2022) 117:647–53. doi: 10.14309/ajg.0000000000001621, 35029166

[15] Arthur S Hannes Philipp N Michael Q. Endoscopic R1/Rx resection of T1 colorectal cancer-what next? Am J Gastroenterol. (2022) 117:603–4. doi: 10.14309/ajg.0000000000001670 35103021

